# Cryptic invasion suggested by a cytogeographic analysis of the halophytic *Puccinellia distans* complex (Poaceae) in Central Europe

**DOI:** 10.3389/fpls.2023.1249292

**Published:** 2023-10-19

**Authors:** Pavel Kúr, Thomas Gregor, Michaela Jandová, Attila Mesterházy, Juraj Paule, Soňa Píšová, Kristýna Šemberová, Petr Koutecký, Michal Ducháček, Gerald M. Schneeweiss

**Affiliations:** ^1^ Department of Biology, Faculty of Science, Jan Evangelista Purkyně University in Ústí nad Labem, Ústí nad Labem, Czechia; ^2^ Department of Botany and Molecular Evolution, Senckenberg Research Institute and Natural History Museum Frankfurt, Frankfurt am Main, Germany; ^3^ Institute of Botany, Czech Academy of Sciences, Průhonice, Czechia; ^4^Independent Researcher, Celldömölk, Hungary; ^5^ Botanischer Garten und Botanisches Museum Berlin, Freie Universität Berlin, Berlin, Germany; ^6^ Department of Forest Biodiversity and Nature Conservation, Austrian Research Centre for Forests, Vienna, Austria; ^7^ Department of Botany, Faculty of Science, Charles University, Prague, Czechia; ^8^ Department of Botany, Faculty of Science, University of South Bohemia, České Budějovice, Czechia; ^9^ Department of Botany, National Museum, Prague, Czechia; ^10^ Department of Botany and Biodiversity Research, University of Vienna, Vienna, Austria

**Keywords:** cryptic invasion, cytogeography, flow cytometry, genetic pollution, halophyte, polyploidy, *Puccinellia distans* agg.

## Abstract

**Introduction:**

Despite the wealth of studies dealing with the invasions of alien plants, invasions of alien genotypes of native species (cryptic invasions) have been vastly neglected. The impact of cryptic invasions on the biodiversity of plant communities can, however, be significant. Inland saline habitats and halophytes (i.e., salt-tolerant plant species) are especially threatened by this phenomenon as they inhabit fragmented remnants of largely destroyed habitats, but at the same time some of these halophytic species are rapidly spreading along salt-treated roads. To study potential cryptic invasion of halophytes, the patterns of genome size and ploidy variation in the *Puccinellia distans* complex (Poaceae), the most rapidly spreading roadside halophyte in Central Europe, were investigated.

**Methods:**

DNA flow cytometry with confirmatory chromosome counts were employed to assess ploidy levels of 1414 individuals from 133 populations of the *P. distans* complex. In addition, climatic niche modelling was used to predict the distributions of selected cytotypes.

**Results:**

Eight groups differing in ploidy level and/or genome size were discovered, one diploid (2*x*; 2*n* = 14), two tetraploid (4*x*A, 4*x*B; 2*n* = 28), one pentaploid (5*x*; 2*n* = 35), three hexaploid (6*x*A, 6*x*B, 6*x*C; 2*n* = 42), and one heptaploid (7*x*; 2*n* = 49). The hexaploids (mostly the 6*x*C cytotype) were widespread through the study area, spreading intensively in both anthropogenic and natural habitats and probably hybridizing with the natural habitat dwelling tetraploids. In contrast, the non-hexaploid cytotypes rarely spread and were predominantly confined to natural habitats.

**Discussion:**

The extensive spread of the hexaploid cytotypes along roadsides has most likely facilitated their incursion into natural habitats. The colonization of new natural habitats by the hexaploids may pose a threat to the indigenous *Puccinellia* populations by compromising their genetic integrity and/or by outcompeting them.

## 1 Introduction

The Poaceae family represents an ecologically and economically important group of plants (Chapman, 1996; Harris, 2014). It is also infamous to invasion ecologists as it contains some important invasive and expansive species such as *Arundo donax* L., *Calamagrostis epigejos* (L.) Roth, *Imperata cylindrica* (L.) P. Beauv., or *Phragmites australis* (Cav.) Trin. ex Steud. (Saltonstall, 2002; Daneshgar et al., 2008; Hardion et al., 2014; Ainouche and Gray, 2016; Marcinkowska-Ochtyra et al., 2018). At the same time, the Poaceae is one of the most taxonomically challenging families of vascular plants, with processes like polyploidization, apomixis and hybridization frequently impeding its study (Keeler, 1998; Hodkinson, 2018).

One of those rapidly expanding grass taxa is the weeping alkali grass, *Puccinellia distans* agg. It is a taxonomic aggregate comprising several halophytic species indigenous to the Atlantic seacoast and/or to inland saline habitats in Europe (Conert, 1994). It is, however, rapidly spreading along de-icing salt-treated roads where it frequently forms monodominant communities in narrow strips along road verges (Hetzel, 2006; Šerá, 2011; Ehl et al., 2019).

The uncontrollable expansion of *P*. *distans* agg. is of great conservation concern. Wetland habitats are suffering from multiple threats including global eutrophication, climate change, immediate habitat destruction, and land use change (Mooij et al., 2005; Petsch, 2016; Šumberová et al., 2021; Kúr et al., 2021). Among them, inland saline habitats have been especially destroyed in Central Europe during the 20^th^ century and they persist in just a few isolated areas. Even the existing localities are threatened by ecological succession, changes in the water regime, or undesirable industrial activities (Bellstedt et al., 2005; Dítě et al., 2009 ; Eliáš et al., 2013; Deák et al., 2014). *Puccinellia distans* (Jacq.) Parl. (its native populations), like many other halophytes, is therefore classified as an endangered species in some countries (Simon and Rühl, 2007; Anonymous, 2008; Grulich, 2012; Opitz, 2019). The origin of the expanding roadside populations, however, has not been investigated so far. Potential invasions of alien genotypes (so called cryptic invasions; Saltonstall, 2002) into indigenous populations of the same species are difficult to detect. They represent yet an underestimated threat that may have a detrimental impact on the biodiversity of native communities, e.g., by genetic assimilation of indigenous genotypes or their demographic swamping (i.e., outcompeting by a fitter genotype; Levin et al., 1996). The expansion of *P*. *distans* agg. therefore poses a potential threat for Central European inland halophytic communities, and especially for native populations of this species complex. Thus, detection of the cryptic invasion is crucial for the conservation of these threatened halophytic communities.

An initial insight into the variation patterns of *P*. *distans* agg. can be gained by studying its cytotype diversity. So far, three ploidy levels have been reported in the *P*. *distans* agg. – diploid (2*n* = 14), tetraploid (2*n* = 28), and hexaploid (2*n* = 42). The information on the distributions of particular cytotypes, however, is very sketchy. There are scattered records for hexaploids from across Europe (Hughes, 1976; Měsíček and Javůrková-Jarolímová, 1992; Conert, 1994; Moravcová et al., 2001), while tetraploids have been rarely reported from Eastern Hungary (Moravcová et al., 2001) and Central Germany (Paule et al., 2017), and diploids have been only recently discovered in Germany (Paule et al., 2017). Any detailed karyologic survey of the *P*. *distans* agg. is, however, lacking. Also, most of the existing karyological studies focus on natural habitats only, while information on cytotype composition of the expanding roadside populations of *P*. *distans* agg. does not exist. Considering that polyploids tend to be better colonizers (Pandit et al., 2011), it may be expected that roadside populations mainly or even exclusively comprise higher-ploidy cytotypes. Natural habitats, on the other hand, may host relict lower-ploidy lineages, and the presence of higher-ploidy cytotypes may indirectly indicate colonization and possible threat by non-indigenous cytotypes.

Cytotype variation in *P*. *distans* agg. is also relevant taxonomically. In Central Europe, the taxonomically challenging complex of *P*. *distans* comprises four main species: *P*. *distans* s. str., *P*. *limosa* (Schur) Holmb., *P*. *peisonis* (Beck) Jáv., and *P*. *fontana* (Portal) Amarell & T. Gregor (Englmaier, 1982; Moravcová et al., 2001; Amarell and Gregor, 2021). Several other, less accepted species have also been described (*P*. *intermedia* (Schur) Janch., Schur, 1866; *P*. *pannonnica* (Hack.) Holmb., Hackel, 1902; *P*. *salinaria* (Simonk.) Holmb., Holmberg, 1920). Morphological discrimination of the species is challenging as there are inconsistencies in their descriptions among different authors, partly probably caused by the great phenotypic plasticity of the species, which results in a large proportion of misidentification by various botanists (Englmaier, 1982; Dítě et al., 2009). Although the species have been considered differing in their ploidy levels (*P*. *distans* and *P*. *fontana* considered hexaploid, and *P*. *limosa* and *P*. *peisonis* considered tetraploid; Englmaier, 1982; Conert, 1994; Amarell and Gregor, 2021), no large-scale study investigating the relationship between cytotype and taxonomic diversity in *P*. *distans* agg. has been carried out so far. Therefore, we intentionally avoid using taxonomic names other than *P*. *distans* agg. in the present study as taxonomic investigation is beyond its scope. However, detailed knowledge of the cytotype variation in *P*. *distans* agg. will be important for future taxonomic studies in this group.

In this study, we address the following questions: (1) What is the cytotype structure of populations of *P*. *distans* agg. in Central Europe? (2) What are the present distribution patterns and potential ecological niches of particular cytotypes? (3) Which cytotypes spread in anthropogenic habitats?

## 2 Materials and methods

### 2.1 Field sampling

A total of 133 populations of *P*. *distans* agg. were sampled during 2018–2020. The main study area where the majority of the populations were sampled spanned Central and partly Eastern Europe. A few additional populations for comparison purposes were sampled in Southern Europe (see **Supplementary Table 1** for a complete list of localities). Usually, 15 individuals per population were collected. Plants were collected at least 1 m apart to minimize repeated sampling of the same genotype. Herbarium vouchers are deposited in the herbarium WU.

For the purpose of statistical evaluation, the localities were categorized into (semi)natural and anthropogenic. As the distinction between purely natural and seminatural habitats was sometimes blurry, and most of the sites are indeed under long-term human management (such as pastures), we took a pragmatic approach and classified all habitats occurring in the regions with known historical occurrences of halophytes and having an extensive management as natural. Thus, the status of natural localities was assigned not only to clearly natural sites like shores of saline lakes, but also to saline meadows, pastures and similar habitats. Anthropogenic habitats included mainly roadsides (including agricultural roads) but also salt mines, salt processing sites, and dump sites.

### 2.2 Flow cytometry

Genome size of all collected plants was determined using flow cytometry. Only fresh tissues were used. The simplified two-step procedure of nuclear isolation and staining (Otto, 1990) modified for plant tissues (Doležel et al., 2007) was employed. Fluorescence intensity of 3500 particles was analyzed using a Partec CyFlow ML flow cytometer (Sysmex, Münster, Germany) equipped with a 532 nm (green) diode-pumped solid-state laser (100 mW output), employing propidium iodide (PI) as a fluorescent stain. *Bellis perennis* (2C = 3.38 pg; Temsch et al., 2021) was used as an internal standard. Each plant was measured individually.

The resulting histograms were evaluated using FloMax 2.52 (Sysmex, Münster, Germany), recording mean fluorescence and coefficient of variation for all fluorescent peaks. Only analyses with coefficients of variation below 2.5% were accepted for further statistical analyses as intra-cytotype variation was detected (see Results) and any suboptimal measurements would easily obscure the results. Genome size (GS, ratio of the mean fluorescence of the sample to the mean fluorescence of the internal standard multiplied by the genome size of the internal standard) was then calculated for each sample. Samples were clustered based on GS using the finite mixture Gaussian distribution modelling in R 4.1.2 (R Development Core Team, 2022). The FitGMM function from the package MGMM (McCaw, 2021) was used. Two values of the number of components (number of groups modelled, i.e., parameter *k*) were tested, namely eight and nine, and the maximum number of iterations (parameter *maxit*) was set to 10^6^. All other parameters were left at their default values.

### 2.3 Chromosome counting

To calibrate the FCM results, chromosomes were counted for 39 individuals representatively covering all the genome size groups discovered. Protocols of Pijnacker and Ferwerda (1984) and Belyayev et al. (2018) were used, with minor modifications. Fresh root tips of pot-cultivated plants were pretreated in ice cold water for 24 h and fixed in fresh fixative solution (pure ethanol and glacial acetic acid 3:1, v/v). The fixed material was stored in the fixative solution at -24°C. The fixed root tips were rinsed in double distilled water (ddH_2_O; 2 × 5 min.) and citric buffer (10 mM sodium citrate, pH 4.8, 1 × 5 min.). Roots were subsequently treated in 0.3% (w/v) enzymatic solution [0.3% (w/v) cellulase, 0.3% (w/v) cytohelicase and 0.3% (w/v) pectolyase, Sigma St. Louis, MO, USA, in 10 mM citric buffer] and incubated in humid chamber for 1 h at 37°C. Digested roots were transferred into ddH_2_O and kept at 4°C. Chromosome preparations were made by smear method (Pijnacker and Ferwerda, 1984). The root meristems were carefully transferred on clean slides and stirred by needle in 40 µL of 75% acetic acid on a warm plate (49°C) for 3 min., fixed in 300 µl fixative solution, washed in pure ethanol and air-dried. Multiple cells per specimen (at least three) were counted. Metaphase plates and chromosome counts were observed by Zeiss Axio Imager.Z2 microscope system at a magnification of 1000*x*. A few representative plates were photographed, and final figures were prepared using Adobe Photoshop version 21.1.3.

### 2.4 Climatic Niche Modelling

For the three main cytotypes (diploids, tetraploids, and hexaploids; see Results), we predicted their potential occurrences in unsampled regions of the main study area and identified any climate-driven limits of their distributional ranges using climatic niche modelling. The maximum entropy modelling approach implemented in Maxent 3.4.1 was employed (Phillips and Dudik, 2008) using the R package *wallace* (Kass et al., 2018). Nineteen bioclimatic variables from WorldClim (Fick and Hijmans, 2017) were downloaded in the highest available resolution (30 arc seconds ≈ 1 km^2^). Spearman correlation coefficients between all pairs of the bioclimatic variables were calculated, and only one variable was retained from each group of highly correlated (|r|>0.7) variables (i.e., bio01, bio02, bio03, bio04, bio06, and bio12). Duplicate data were removed by spatial thinning at a 1 km distance, and 10,000 background points were randomly sampled without replacement from the study area (the study area was defined as a minimum convex polygon around all the sampling sites in the main study area, expanded by a buffer zone of 2 degrees). The occurrences were partitioned into testing and training bins using the jackknife method. The maxent models were run with the regularization multiplier (RM) values ranging from 0.5 to 10 (incremented by 0.5) and five alternative settings for feature classes (i.e., types of functions used to fit the response curves; Merow et al., 2013) and their combinations (i.e., L, LQ, H, LQH, LQHP, where L = linear, Q = quadratic, H = hinge, and P = product; see Phillips et al., 2006; Phillips and Dudik, 2008). The Akaike information criterion (AIC; Bozdogan, 1987) was used to select the model with the highest predictive power.

## 3 Results

### 3.1 Ploidy levels and chromosome counts

**Figure 1:**
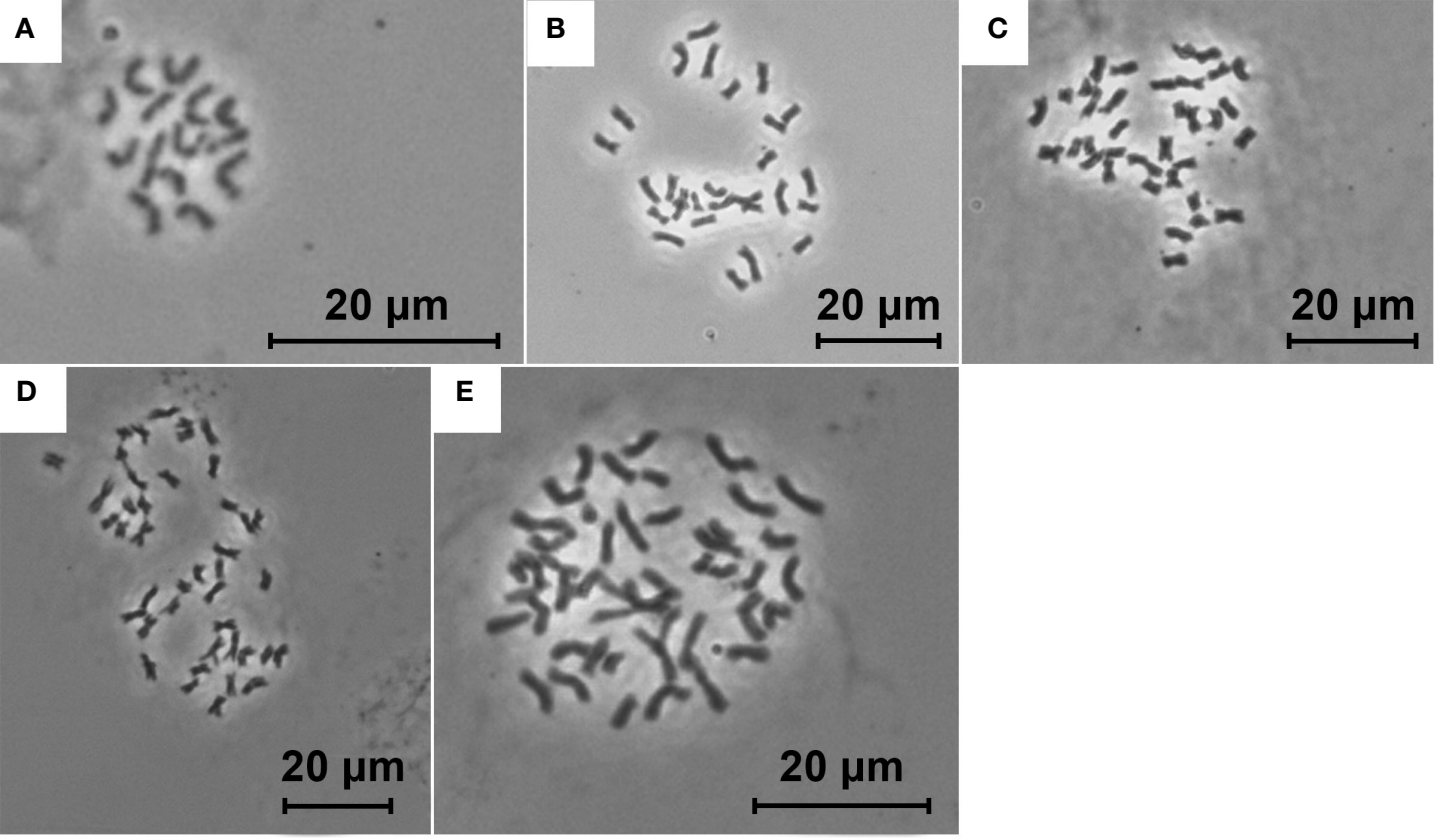
Mitotic chromosome spreads of diploid [**(A)** 2*n* = 14], tetraploid [**(B)** 2*n* = 28], pentaploid [**(C)** 2*n* = 35], hexaploid [**(D)** 2*n* = 42], and heptaploid [**(E)** 2*n* = 49] *Puccinellia distans* agg.

**Figure 2:**
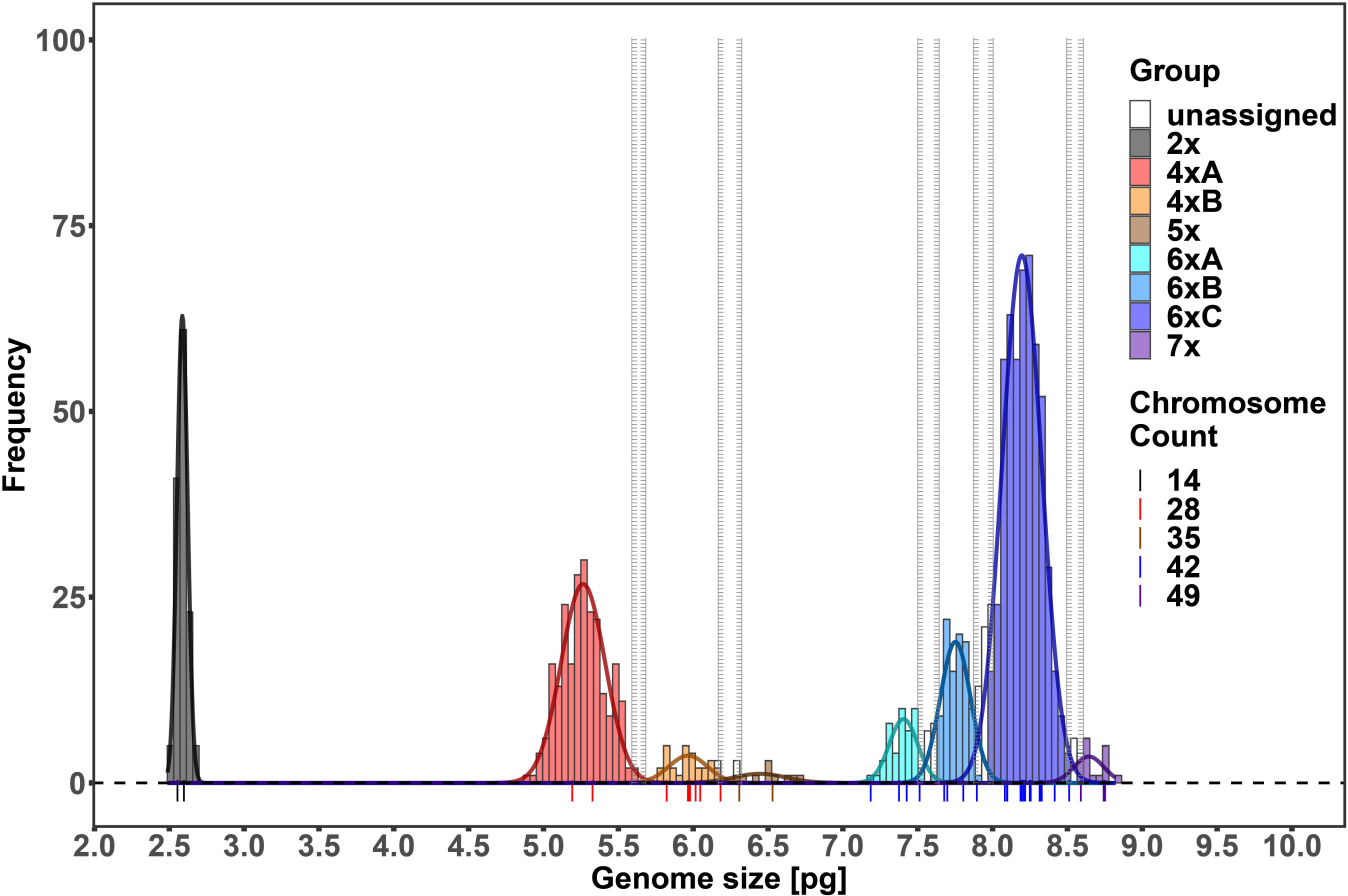
Histogram of the genome sizes of the analyzed *Puccinellia distans* agg. samples. The curves show the fitted finite mixture Gaussian distributions for k = 8. Individual genome size groups are depicted in different colors. Colored symbols represent the positions of individuals with counted chromosomes.

In total, 1414 FCM samples were measured, 1153 of which met the CV cut-off criterion (see **Supplementary Table 2** for an overview of all the measured samples). Raw flow cytometric data are available at the Zenodo digital repository (Kúr et al., 2023). Chromosome counting confirmed the presence of five distinct ploidy levels, i.e. diploid (2*n* = 14), tetraploid (2*n* = 28), pentaploid (2*n* = 35), hexaploid (2*n* = 42), and heptaploid (2*n* = 49) (**Figure 1**). The finite mixture Gaussian distribution modelling of the GS for k = 8 found eight clear clusters, delimiting two clusters within the tetraploids and three clusters within the hexaploids (**Figure 2**, **Table 1**; see **Supplementary Figure 1** for a chart of the raw C-values). Running the same procedure for k = 9 did not produce interpretable results as it distinguished one more group among the tetraploids which, however, was very small and entirely overlapped with the other fitted group (see **Supplementary Figure 2** for the results of the finite mixture Gaussian modelling for k = 9). Hence, we used the clusters fitted for k = 8 for the definition of the GS groups used for further analyses. The GS groups were delimited based on strict criteria: (1) boundaries between overlapping heteroploid groups were defined by the individuals with highest/lowest GS that had counted chromosomes; (2) boundaries between overlapping homoploid groups were defined as the 1^st^ and 99^th^ percentiles of the corresponding Gauss curves. Individuals not falling within the defined ranges were left as unassigned. The result was one diploid (2*x*), two tetraploid (4*x*A, 4*x*B), one pentaploid (5*x*), two hexaploid (6*x*A, 6*x*B, 6*x*C), and one heptaploid (7*x*) groups (**Figure 2**).

**Table 1:** Genome size estimates for particular genome size groups (inferred from the finite mixture Gaussian distribution model).

Group	Genome Size [pg]	Standard Deviation
**2*x***	2.587	0.036
**4*x*A**	5.265	0.147
**4*x*B**	5.966	0.128
**5*x***	6.445	0.173
**6*x*A**	7.405	0.095
**6*x*B**	7.752	0.101
**6*x*C**	8.196	0.132
**7*x***	8.643	0.100

### 3.2 Population structure and present and modelled geographical distribution of cytotypes

**Figure 3:**
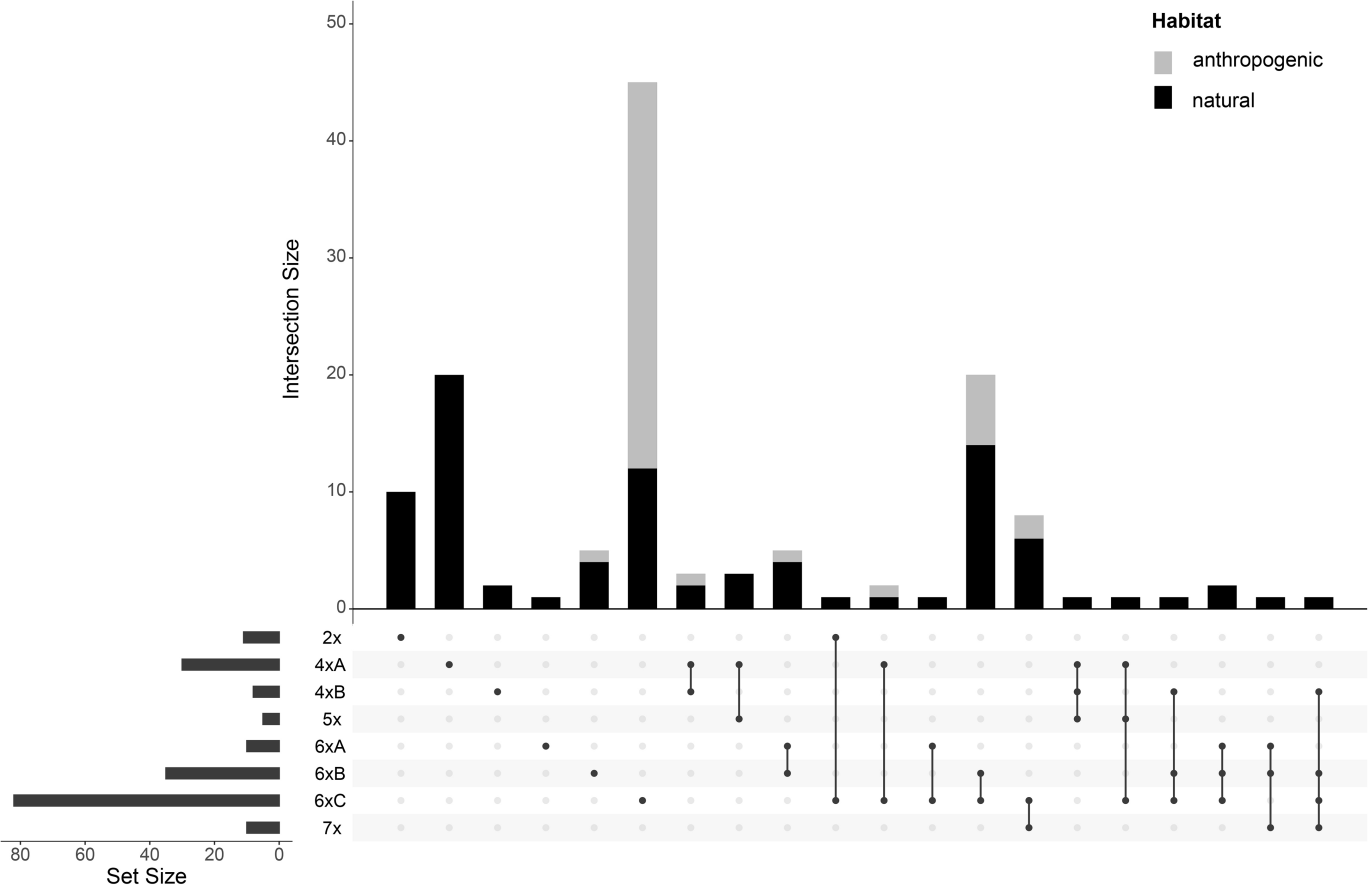
Upset intersection plot (Conway et al., 2017) showing the frequencies of pure and mixed-ploidy populations of *Puccinellia distans* agg. Each row represents a different genome size group, and each bar represents the number of populations with the particular mixture of the genome size groups (denoted by connected black dots under each bar). In addition, the proportions of localities found in anthropogenic and natural habitats are denoted by stacked bars of different colors.

**Figure 4:**
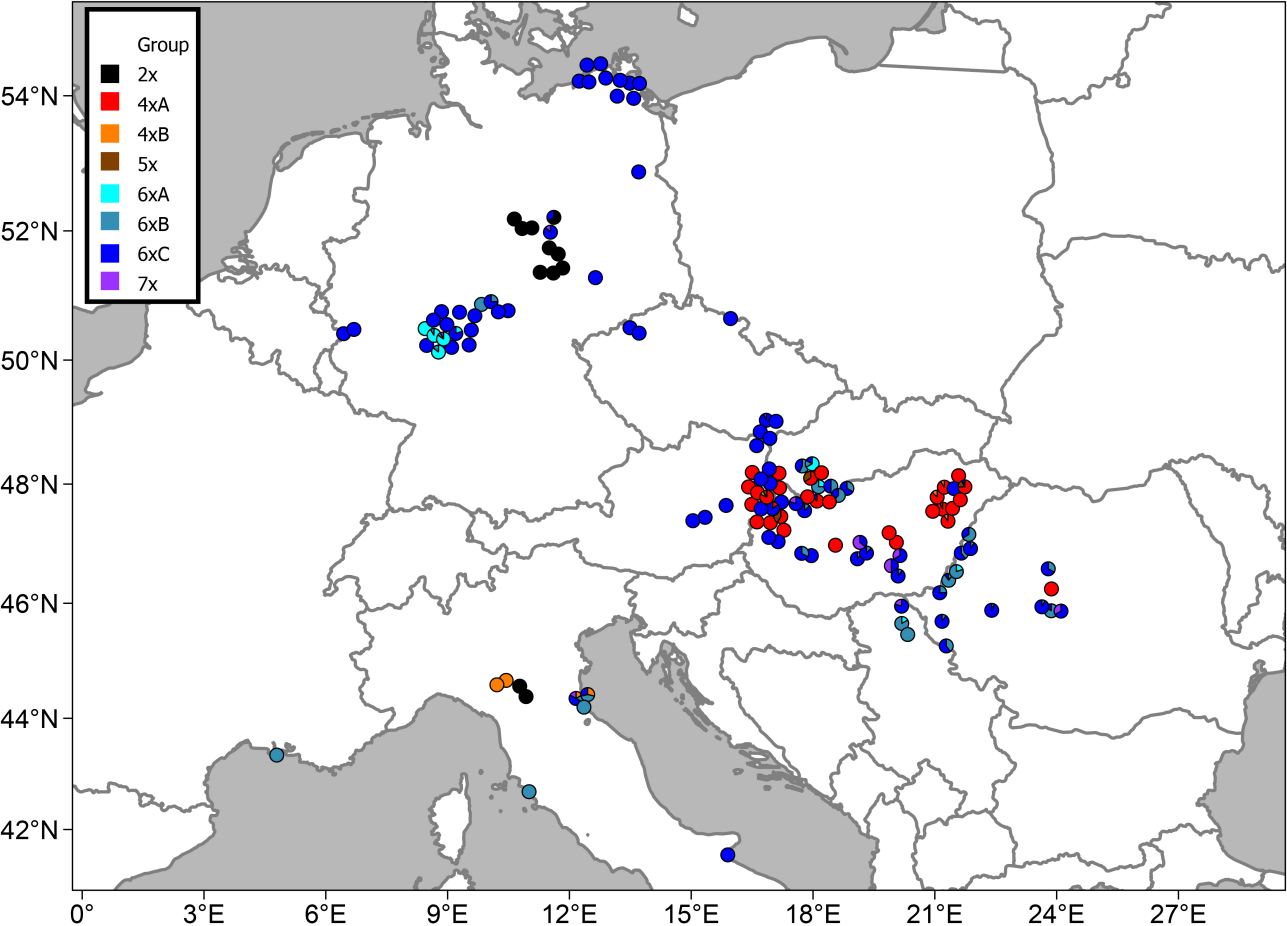
Distribution of the studied populations of *Puccinellia distans* agg. and their cytotype compositions.

**Figure 5:**
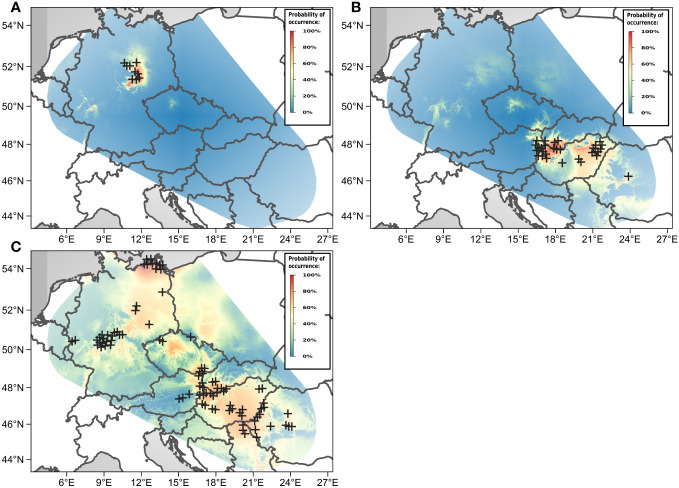
Predicted climatic suitability of habitats for the **(A)** diploid, **(B)** tetraploid, and **(C)** hexaploid cytotypes of *Puccinellia distans* agg. in the main study area. The actual cytotypes’ occurrences are plotted with black crosses.

Thirty-eight percent of the populations were mixed, i.e., containing individuals from different GS groups (**Figure 3**). Diploids (cytotype 2*x*) in the main study area occurred in a confined region in Central Germany. Additionally, two diploid populations were also found in N Italy (**Figure 4**). Suitable climatic conditions for the diploids were predicted mostly in the area of their current distribution (in the main study area), with some minor additions in South-Western Germany and North-Eastern France (**Figure 5**; the best maxent model L, RM = 1, AIC = 185.8, omission rate = 0.125 ± 0.354 [mean ± standard deviation]).

Tetraploids in the main study area were found nearly exclusively in the Pannonian Basin (with one outlying population in the Transylvanian Plateau in Romania) (**Figure 4**). The 4*x*A cytotype clearly prevailed; the 4*x*B occurred only sporadically in mixed populations with the 4*x*A cytotype. Outside the main study area, plants with the GS fitting the 4*x*B cytotype were also found in N Italy. The predicted climatic suitability for the tetraploids highlights mostly the area of their current distribution in the main study area as having the highest occurrence probability (**Figure 5**, the best maxent model LQHP, RM = 1.5, AIC = 647.4, omission rate = 0.038 ± 0.196 [mean ± standard deviation]).

In contrast, hexaploids were ubiquitous through the main study area (**Figure 4**). The most common cytotype was 6*x*C, which typically formed uniform populations (**Figure 3**). The cytotype 6*x*A was relatively rare, confirmed in just 10 localities, and it mostly occurred in mixed populations with 6*x*B and 6*x*C. The cytotype 6*x*B was relatively abundant, again mostly in mixed populations with 6*x*C. Outside the main study area, plants with the GS fitting the 6*x*B and 6*x*C cytotypes were also found in Italy and France. Climatic modelling for the hexaploids showed most of the main study area as suitable, with lower occurrence probabilities only in mountainous regions (**Figure 5**, the best maxent model H, RM = 2.5, AIC = 2200.1, omission rate = 0.025 ± 0.158 [mean ± standard deviation]).

Pentaploids (cytotype 5*x*) were found exclusively in mixtures with other cytotypes, mostly tetraploids, in the Pannonian Basin. Similarly, heptaploids (cytotype 7*x*) were found exclusively in mixed populations, predominantly with hexaploid cytotypes, and they were distributed randomly across the whole study area (**Figures 3**, **4**).

### 3.3 Cytotypes spreading to anthropogenic habitats

There was a clear difference in the cytotype frequency between populations from natural and anthropogenic habitats. A majority of the populations from anthropogenic habitats were hexaploid (with the cytotype 6*x*C clearly prevailing). Tetraploids were also rarely found in anthropogenic habitats (roadsides) in the areas of their natural occurrence (Eastern Hungary, Eastern Austria), as were heptaploids. Diploids and pentaploids were not found in anthropogenic habitats at all (**Figure 3**).

## 4 Discussion

In this study, cytotype distribution and variation in ploidy level and genome size was studied to elucidate the geographical structure of *Puccinellia distans* agg., a rapidly spreading halophyte. We hypothesize that polyploids may be better colonizers, and cytotype population structure may thus indicate cryptic invasions of non-native genotypes into endangered relic lineages.

A polyploid series was found among the analyzed samples of *P*. *distans* agg., ranging from diploids to heptaploids. Whereas the existence of diploids, tetraploids, and hexaploids has been already known in *P*. *distans* agg. in Central Europe (Moravcová et al., 2001; Amarell and Gregor, 2021), pentaploids and heptaploids are reported here for the first time. In Central Europe, these uneven ploidy levels have been so far reported only from the coastal species *P*. *maritima* (Huds.) Parl., a species with a long aneuploid series ranging from 2*n* = 14 to 2*n* = 112 (Paule et al., 2017).

### 4.1 Origin of cytotypes

Multiple homoploid groups differing in the genome size were found among the tetraploids and the hexaploids. Although the boundaries between the groups were not sharp, it is unlikely that the resulting pattern is an artifact as stringent filtering of the FCM analyses was used (only samples with coefficient of variation below 2.5% were kept). Clearly, two groups among the tetraploids (4*x*A and 4*x*B) and three groups among the hexaploids (6*x*A, 6*x*B, and 6*x*C) were present.

The minor (i.e., less frequent) cytotypes 4*x*B, 6*x*A, and 6*x*B were observed nearly exclusively in mixed populations with the respective major (i.e., dominant) cytotypes 4*x*A and 6*x*C, suggesting the minor cytotypes are evolutionarily derived from the major ones (the same seems to be true for the heptaploids which were observed nearly exclusively in the presence of the 6*x*C cytotype). The observed intraploidal GS variation could be caused by the presence of aneuploidy. Polyploid lineages, especially young ones, tend to produce unbalanced gametes, which may be viable and produce aneuploid offspring (Ramsey and Schemske, 2002). However, our data does not support this scenario in the case of the tetraploid and hexaploid *P*. *distans* agg. as we did not find any aneuploids in the chromosome counts of any of the 39 individuals counted, including the 15 individuals of the minor cytotypes (**Supplementary Table 2**). This finding is congruent with the general lack of aneuploidy in the genus *Puccinellia*, with only *P*. *maritima* being the exception (Hughes, 1976; Rice et al., 2015). Thus, a more probable scenario is that the minor cytotypes are generated via the segregation of homeologous chromosomes of different sizes in the parental cytotypes. This would require the 4*x*A and 6*x*C cytotypes to be of allopolyploid origin, which may well be the case. This mechanism explaining intraspecific genome size variation was suggested for example in *Festuca pallens* Host and is most likely to occur in young rapidly radiating groups (Šmarda and Bureš, 2010).

The most probable pathway of the formation of the pentaploids is a cross between tetraploids and hexaploids as no aneuploids or triploids, which could otherwise enable their formation, were found. The pentaploids were indeed found exclusively in mixed populations with tetraploids and/or hexaploids, and they were discovered exclusively in regions with a common co-occurrence of both tetraploids and hexaploids (i.e., the Pannonian Basin), which further corroborates this scenario.

### 4.2 Geographic distribution of cytotypes

Contrary to the conclusions of Moravcová et al. (2001), who reported tetraploids of *P*. *distans* agg. only from Eastern Hungary, we discovered tetraploid cytotypes in an extensive area ranging from Eastern Austria and Southern Slovakia through Hungary to Romania. The most likely reason of the discrepancies with the literature is an unintended bias in the sampling of Moravcová et al. (2001) who missed the tetraploids’ localities in the western part of their study area.

On the other hand, our study did not discover any tetraploids in Central Germany as reported in Paule et al. (2017). There are three possible explanations of this result. First, there might be insufficient sampling in our study. A second possible explanation is that the tetraploid records from Central Germany are wrong. Third, a cytotype turnover (i.e., a replacement of one cytotype by another due to selective pressure), as observed for example by Trávníček et al. (2010) in *Vicia cracca* L., could also explain the observed discrepancy. While an unintentional oversight of tetraploids by us is possible, it is unlikely as our sampling in the area of alleged tetraploids’ occurrence was fairly thorough (18 localities), and we even had material from one exact locality where tetraploids were previously reported (Bad Salzungen). Paule et al. (2017) already suggested the tetraploid counts of *P*. *distans* agg. from Germany, all reported by Link (1992), to be incorrect. These counts were recorded in scope of a diploma thesis, and based on the provided photographic documentation it is difficult to interpret them as tetraploid. So, although we cannot fully exclude a historical presence of a tetraploid cytotype of *P*. *distans* agg. in Central Germany (a critical inspection of the published records, possibly including revisions of any existing herbarium material, would be useful), it seems currently more likely, given the absence of any other sources corroborating the findings of Link (1992), that the tetraploid *P*. *distans* agg. cytotypes have never been present in the German inland.

Diploids, in accord with the already known occurrences (Amarell and Gregor, 2021), were confirmed in a small area in Central Germany. Climatic niche modelling supported a general habitat unsuitability in other regions. Outside the main study area, diploid populations were also found in N Italy. Although in the latest edition of Flora d’Italia (Pignatti et al., 2017), *P*. *distans* is described as having diploid, tetraploid, pentaploid, and hexaploid cytotypes, we were unable to find any original publications reporting chromosome counts of *P*. *distans* agg. from Italy, and it is not clear whether the mentioned chromosome counts come from Italian populations at all. There are only a few published records of diploid *Puccinellia* from the European inland: Avdulow (1931) – European part of Russia, Montserrat and Montserrat (1988) – Spain, and Amarell and Gregor (2021) – Germany. Therefore, the diploids from Italy, found so far only in two close localities of mud volcanos, probably represent the first verifiable report of diploids from this country. The great cytotype diversity observed in the eight measured Italian localities alone also suggests that the patterns of diversity in *P*. *distans* agg. in Southern Europe may be as complex as in Central Europe, thus deserving a separate study.

### 4.3 Cytotypes spreading to anthropogenic habitats

The prevalence of the hexaploids (especially the 6*x*C cytotype) in anthropogenic habitats suggests their high propensity to adventive spread. Judging from their observed occurrences in localities far away from any natural habitats, it seems that the hexaploids are capable of an efficient long-range dispersal. It is therefore likely that a vast majority of literary reports of *P*. *distans* from road verges in Europe (Hetzel, 2006; Šerá, 2008; Šerá, 2010; Šerá, 2011) is attributable to the hexaploids. It is also likely that the hexaploid cytotypes have pervaded many natural habitats. We have detected three mixed-ploidy hexaploid-tetraploid populations and one hexaploid-diploid population in the main study area, 75% of which were in natural habitats. Although the hexaploids in the natural habitats might potentially represent local native genotypes, it is more probable that, at least in some cases, the widespread hexaploids represent an invading lineage in the early stage of its incursion into natural habitats. Alarmingly, indirect indications of the hybridization between the tetraploids and the hexaploids in the form of the presumably hybridogenous pentaploid cytotype exist.

The low expansion potential of the non-hexaploid cytotypes can most probably be attributed to their different life histories. Their lower vigor compared to the hexaploids could most probably manifest in lower drought and temperature resistance and/or lower seed set (Moura et al., 2021). Polyploidy has been indeed demonstrated as an important determinant of invasiveness/expansiveness in plants (te Beest et al., 2012; Walczyk, 2018; Moura et al., 2021). Our climatic models show a low habitat suitability in areas beyond the current distributions of the diploids and tetraploids. Although climatic models have their limitations (Oliveira et al., 2021) and other than climatic factors certainly play a role in the cytotypes’ distributions, it appears unlikely that these cytotypes can spread much outside their current distribution areas and therefore pose any threat to other native populations.

## 5 Conclusions

Halophytes, inhabiting stressful habitats, face many challenges ranging from global climatic and edaphic changes to direct habitat destruction due to e.g., industrial activities (Bellstedt et al., 2005). A neglected threat represents cryptic invasion, when a migration of a non-native genotype is facilitated by the salt-treated road network connecting many previously isolated localities hosting relic populations. In congruence with the fact that polyploidy is largely acknowledged as a preadaptation for colonization of new habitats (te Beest et al., 2012), higher polyploids (mostly hexaploids) were dominant in the anthropogenic habitats. The reproductive barriers are generally lower between higher-than-diploid ploidies (Sutherland and Galloway, 2017) and the detected pentaploid cytotype may indicate a gene flow between the non-native (hexaploid) and native (tetraploid) lineages. The current results raise the awareness about the threat of cryptic invasion followed by hybridization and potential outcompeting of the native lineages. Future research should thus elucidate the genetic variation in the *P*. *distans* agg. and focus on detecting and classifying the impact of cryptic invasion on the variation within the *P*. *distans* agg. lineages.

## Data availability statement

The datasets presented in this study can be found in online repositories. The names of the repository/repositories and accession number(s) can be found below: https://zenodo.org/record/8077314.

## Author contributions

TG, AM, MD and JP contributed to the conception and design of the study and helped with the field sampling. MJ did chromosome counting. SP and KS took care of the cultivation of plant material. PKo provided assistance with the flow cytometric analyses. PKú initialized the study and wrote the first draft of the manuscript. GS advised on the final version of the manuscript. All authors contributed to the article and approved the submitted version.

## Supplementary material

Supplementary Figure 1 Scatterplot of the genome sizes of the analyzed *Puccinellia distans* agg. samples ordered by increasing genome size.

Supplementary Figure 2 Finite mixture Gaussian model for k = 9.
