# Supplementary material for: Cryptic invasion suggested by a cytogeographic analysis of the halophytic Puccinellia distans complex (Poaceae) in Central Europe
Source: Front Plant Sci. 2023 Oct 19;14:1249292. doi: 10.3389/fpls.2023.1249292 (PMC10620967; doi:10.3389/fpls.2023.1249292)
Supplement: Supplementary Figure 2 — Finite mixture Gaussian model for k = 9. [file Image_2.pdf]

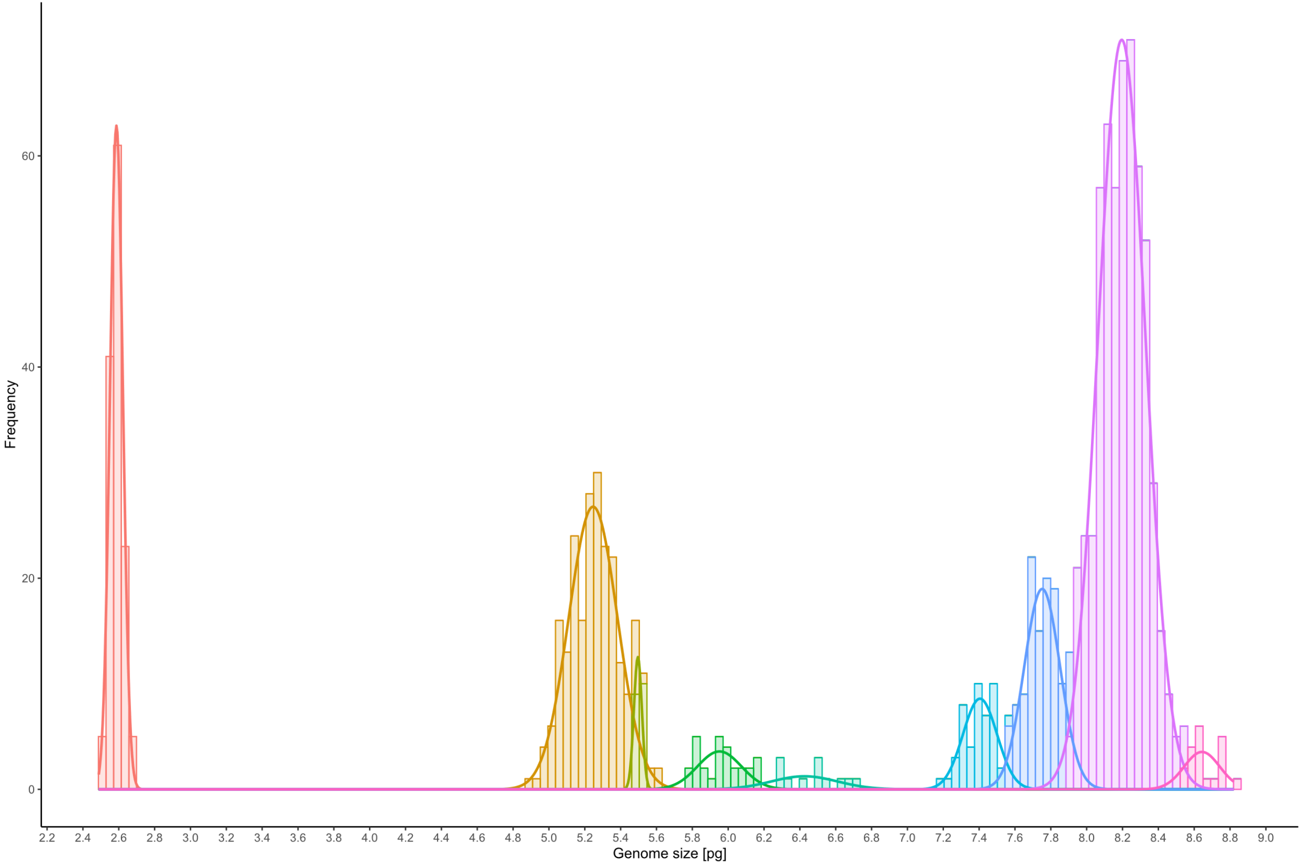

Supplementary Figure 2. Histogram of the genome sizes of the analyzed *Puccinellia distans* agg. samples. The curves show the fitted finite mixture Gaussian distributions for k = 9. Individual genome size groups are depicted in different colors.
